# Effects of two *Bacillus velezensis* strains isolated from different sources on the growth of *Capsicum annum*

**DOI:** 10.3389/fmicb.2024.1504660

**Published:** 2024-12-09

**Authors:** Bao Wang, Shimeng Tan, Mingde Wu, Yujie Feng, Wanrong Yan, Qian Yun, Xuncong Ji, Runmao Lin, Zhixiang Zhao

**Affiliations:** ^1^Key Laboratory of Plant Disease and Pest Control of Hainan Province, Institute of Plant Protection Academy of Agricultural Sciences (Research Center of Quality Safety and Standards for Agricultural Products of Hainan Academy of Agricultural Sciences), Haikou, China; ^2^Key Laboratory of Green Prevention and Control of Tropical Plant Diseases and Pests, Ministry of Education, School of Tropical Agriculture and Forestry, School of Breeding and Multiplication (Sanya Institute of Breeding and Multiplication), Hainan University, Haikou and Sanya, China; ^3^School of Life and Health Science, Hainan University, Haikou, China; ^4^Key Laboratory of Plant Pathology of Hubei Province, Huazhong Agricultural University, Wuhan, China

**Keywords:** *Bacillus velezensis*, antifungal activity, biological control, plant growth-promoting microorganisms, soil rhizosphere community, metagenomic

## Abstract

Microbial inoculants offer an environmentally friendly approach to enhance plant growth and control disease. In this study, two *Bacillus velezensis* strains, HKSSLJEBR3 (R3) and Ya-1 were isolated from pepper plant roots and tropical rainforest soil, respectively. Both strains exhibited strong antifungal activity against *Fusarium oxysporum* f. sp. *capsici*, with inhibition rates of 48.54 ± 0.66% for R3 and 49.35 ± 1.44% for Ya-1. In greenhouse trials, R3 significantly boosted pepper growth, with a 22.12% increase in plant height, 46.44% more leaves, and 56.29% greater fresh weight. These enhancements were likely due to the strong affinity between R3 and pepper plants. Both strains also improved soil quality, with R3 increasing available potassium (AK) by 5.13% and soil organic matter (SOM) by 4.03%, while Ya-1 showed more significant increases. Metagenomic analysis revealed that both strains altered the rhizosphere microbiome, with R3 promoting *Pseudomonas* and suppressing *Fusarium*. These results suggest that the R3 strain has strong potential for enhancing pepper growth, improving soil health, and reshaping the rhizosphere microbiome.

## 1 Introduction

Pepper plant (*Capsicum annum* L.) is one of the main horticultural crops in the world. However, biotic and abiotic stresses significantly affect pepper plant growth. For example, Fusarium wilt of pepper, a stubborn soil-borne disease caused by *Fusarium oxysporum*, seriously limits the development of the pepper industry (Shaheen et al., 2021). In addition, long-term abuse of chemical fertilizers leads to environmental pollution, obstacles to continuous cropping, and reduction of soil microbial diversity (Geisseler and Scow, 2014; Bai et al., 2020). As a promising alternative, microorganisms offer an eco-friendly and sustainable solution for both disease management and plant growth promotion. For example, peanut stem rot can be controlled by *Streptomyces* sp. RP1A-12 to promote peanut growth (Jacob et al., 2018). *Trichoderma asperellum* FJ035 can be employed to control cucumber Fusarium wilt through antagonism and spatiotemporal competition with *Fusarium oxysporum* (Wang et al., 2023).

Soil is a complex and dynamic environment, where the rhizosphere microorganisms play a pivotal role in regulating plant growth and maintaining agroecosystem health (Hakim et al., 2021; Khan et al., 2021). These microorganisms, which include bacteria, fungi, archaea, and viruses, are essential for nutrient cycling, disease suppression, and plant development. However, the majority of these microbes cannot be cultured *in vitro*, presenting a significant challenge to understanding their roles in the soil. The advent of metagenomic sequencing technologies enables the capture of genomic information from the entire microbial community present in soil. This technology offers valuable insights into microbial diversity and community structure, helping us better understand how these microbes contribute to soil health and plant growth (Daniel, 2005; Nwachukwu and Babalola, 2022; Nam et al., 2023).

Plant growth-promoting microorganisms (PGPM) offer an effective approach to addressing global challenges such as food security, soil degradation and environmental sustainability. PGPM inoculants can change the microbial community in the plant rhizosphere, improving the soil’s fertility and environment as well as crop growth (Xu et al., 2022; Zhou et al., 2022; Tian et al., 2023). *Bacillus velezensis* (*B. velezensis*) is one of the most studied PGPM as a biological control agent due to its wide distribution, fast reproduction, and strong stress resistance (Ye et al., 2018). The biocontrol mechanisms of *B. velezensis* include the production of antimicrobial substances, bioactive enzymes, and volatile organic compounds, the stimulation of induced systemic resistance, and the promotion of plant growth (Rabbee et al., 2019, 2023). For instance, Torres et al. isolated a strain of *B. velezensis* XT1 from saline–alkali soil that increases the aerial fresh weight of tomato, pumpkin, cucumber, and pepper plants (Torres et al., 2020). Choub et al. demonstrated that the *B. velezensis* strain CE100 can produce indole-3-acetic acid and possibly ammonium and stabilize phosphate to enhance the availability of essential nutrients (Choub et al., 2021). Wang et al. showed that the *B. velezensis* strain Yb-1 can prevent and control pepper anthracnose in the field (Wang et al., 2023). Currently, most biocontrol microorganisms come from soil or plant tissue. However, the inoculation and effects of biocontrol microbial strains of the same species but from different sources on the same plants are unclear.

This study investigates the effects of two bacterial strains, Ya-1 and R3, on the growth and development of hot peppers. The Ya-1 strain was isolated from the soil of tropical rainforests, while R3 was obtained from the root system of a pepper plant. Preliminary experiments showed that both strains exhibit excellent potential for biocontrol. The two strains were identified using morphological and molecular biological analyses. The study evaluates the effects of these strains on pepper growth, soil physical and chemical properties, and rhizosphere microbes, in order to gain insight into the mechanisms driving growth promotion.

## 2 Materials and methods

### 2.1 Isolation and growth conditions of microorganisms

Soil samples were collected from a tropical rainforest, in Shuiman Township, Wuzhishan City, Hainan Province, China (109°39′51.95″E, 18°54′10.19″N; altitude: 683.8 m) on March 16, 2013. To prepare soil suspension, 1.0 g of soil was mixed with 99 mL of sterile water, and resulting suspension was diluted 10, 100, and 1,000 times. A 100 μL aliquot from each dilution was plated onto Luria Bertani (LB) agar plates (containing 10 g/L tryptone, 5 g/L yeast extract, 10 g/L NaCl, and 20 g/L agar) and cultured at 28°C for 48 h. Colonies were selected based on colony morphology, size, and color, and single colonies were transferred to new LB plates for five successive subcultures to ensure bacterial purity. Strain Ya-1 was selected based on preliminary plate confrontation tests and stored at −80°C in 20% glycerol.

Pepper plant samples were collected from Shishan Town, Haikou City, Hainan Province, China (110°14′32.73″E, 19°45′59.16″N; altitude: 47.6 m) on February 28, 2022. To remove surface impurities, the samples were rinsed with sterile water for 30 s. Pepper root samples (1.0 g) were washed thrice with sterile water, disinfected with 75% ethanol for 1 min, and rinsed three more times with sterile water. The sterilized samples were incubated with 0.1% HgCl_2_ for 1 min, rinsed five times with sterile water, and ground in a sterile mortar containing 9 mL of sterile water. The sample homogenates were diluted in a gradient, coated on LB plates, and incubated at 28°C. After purification and preliminary screening in plate confrontation experiments, the R3 strain was selected and stored at −80°C.

### 2.2 Determining antifungal activity

The pathogenic *F. oxysporum* CMTT4-1 strain was isolated from susceptible pepper plants in Chengmai County, Hainan Province, China and was preserved by the Biological Control Laboratory of the Institute of Plant Protection, Academy of Agricultural Sciences, Hainan Province, China. The strain was identified as *F. oxysporum* f. sp. *capsici* through a combination of morphological and molecular methods. The pathogen was activated by culturing on potato dextrose agar (PDA) (200 g potato, 20 g glucose, and 20 g agar per liter) at 28°C for 7 days. A 6 mm diameter pathogen fungal growth plug was then transferred to the center of a fresh PDA plate. The biocontrol strains R3 and Ya-1 were cultured on LB plates, and single colonies were inoculated into 100 mL of LB liquid medium, where they were inoculated at 30°C and 180 rpm for 24 h. Afterward, 6 mm sterile filter paper disks were placed 3 cm from the pathogen fungal growth plug, and 5 μL of biocontrol bacterial suspension were inoculated onto the sterile filter paper. LB liquid culture medium (5 μL) was inoculated on a filter paper as a control. Each treatment was repeated three times. The pathogen fungal growth plug was cultivated at 28°C. After 7 days, the diameter of the pathogen was recorded, and the antifungal rate was calculated with the following formula (Huang et al., 2023):

Inhibition (%) = [(diameter of untreated pathogen-diameter of treated pathogen)/diameter of untreated pathogen] × 100%.

### 2.3 Identification of biocontrol bacteria from different sources

The biocontrol bacteria were cultured on LB plates at 28°C for 2 days, and the morphology, color, size, and edges of the colonies were observed to morphologically identify the isolates based on the Manual for Systematic Identification of Common Bacteria (Dong and Cai, 2001). To validate the identity of the isolates, molecular identification was carried out by sequencing the 16S rRNA and gyrase subunit A (*gyrA*) genes. Total genomic DNA was extracted from strains R3 and Ya-1 using a bacterial genomic DNA extraction kit (DP302, Tiangen Biochemical Technology Co., Ltd., Beijing, China), according to the manufacturer’s protocol. PCR amplification of the 16S rRNA and *gyrA* genes was performed with the following primers: 16S rRNA 27F/1492R (Dees and Ghiorse, 2001) and *gyrA* F/R (Chun and Bae, 2000). The 30 μL PCR reaction mixture consisted of 1 μL of genomic DNA (100 ng/μL), 15 μL of 2 × Taq PCR Master Mix, 1 μL of each primer pair (10 μM), and 12 μL of ddH_2_O. The amplification program was as follows: 95°C for 5 min, 95°C for 30 s, 55°C for 30 s, 72°C for 1 min (30 cycles), and 72°C for 10 min, followed by a 4°C soak. The PCR products were analyzed by electrophoresis on a 1% agarose gel and purified using a DNA gel recovery kit (AXYDEN, Silicon Valley, United States). The purified PCR products were sequenced by Hainan Nanshan Biotech Co., Ltd. (Haikou, China).

The nucleotide sequences were compared using the BLAST tool of the NCBI database, and the neighbor-joining (NJ) method from MEGA software (Version 11.0, Richlandtown, PA, USA) was used to combine the 16S rRNA gene and *gyrA* gene sequences. A polygenic phylogenetic tree was generated to analyze the taxonomic status of the two strains. The nucleotide sequences were deposited in GenBank for subsequent scientific community access.

### 2.4 Pepper pot experiment

#### 2.4.1 Preparation of pepper seedlings

The pepper variety Xinchangjiao No.8 an F1 hybrid developed by Huaibei City Kubota Seed Co., Ltd. (China), was used in this study. The seedlings were cultivated in COMPO SANA® home gardening nutrient soil (COMPO GmbH, Germany), within seedling pots of 8 cm diameter and 10 cm height. Prior to sowing, the pepper seeds were sterilized in a 5% sodium hypochlorite solution for 10 min, followed by thorough rinsing with sterile water to remove any residual disinfectant. The plants were grown under controlled environmental conditions (25°C, 70% relative humidity, 12 h light/12 h dark photoperiod) until the seedlings reached the four-leaf stage. Sterile water was regularly applied to maintain the moisture content of the growing medium.

#### 2.4.2 Preparation of microbial inoculants

The R3 and Ya-1 strains were inoculated onto LB agar plates and incubated at 28°C for 24 h. Single colonies were subsequently picked and transferred to a 250 mL Erlenmeyer flask containing 100 mL LB liquid medium. The cultures were then incubated at 30°C with shaking at 180 rpm for 12 h to establish the inoculum. A 5 mL aliquot of the inoculum was then transferred into a 1,000 mL Erlenmeyer flask containing 500 mL of fresh LB liquid medium, and the culture was incubated under the same conditions (30°C, 180 rpm) for 24 h. Afterward, the bacterial cells were harvested by centrifugation at 5000 rpm for 10 min, and the pellet was resuspended in sterile water to achieve a final concentration of 1 × 10^6^ CFU/mL.

#### 2.4.3 Treatment of pepper with R3 and Ya-1 inoculants

Pepper seedlings were grown to the four-leaves stage (Section 2.4.1) and subsequently treated with R3 and Ya-1 inoculants via soil drenching (Section 2.4.2). Thirty seedlings were inoculated per treatment, with each treatment repeated three times. A 20 mL dose of bacterial agent was applied per plant, while the control group received sterile water. Inoculations were performed twice, with a 10-day interval between treatments. Plant height, root length, leaf number, and fresh weight were measured 20 days after the second inoculation.

### 2.5 Rhizosphere sampling and measurement of soil physicochemical properties

#### 2.5.1 Collection of rhizosphere soil from pepper plants

Rhizosphere soil samples were collected from the potted peppers 20 days after the final microbial inoculation. The plants were carefully uprooted, and large soil aggregates were removed. The roots were shaken to remove loosely attached soil, and the remaining soil was collected from the roots using a sterile brush. The soil samples were then stored at −80°C. A total of six rhizosphere soil samples per treatment were selected for genomic DNA extraction and metagenomic sequencing.

#### 2.5.2 Determination of soil properties

Soil samples (500 g per treatment) were collected from potted plants 20 days after the final microbial treatment. The samples were dried in a ventilated area. Total phosphorus (TP) and available phosphorus (AP) were measured according to the forestry industry standard LY/T1232-2015, while available potassium (AK) was determined following LY/T1234-2015 (LY/T1232, 2015; LY/T1234, 2015). Total nitrogen (TN) was quantified according to the agricultural industry-standard NY/T1121.24–2012, and soil organic matter (SOM) was assessed based on NY/T1121.6–2006 (NY/T1121.6, 2006; NY/T112.24, 2012). Each treatment was repeated six times.

### 2.6 DNA extraction and metagenomic sequencing

For metagenomic analysis of rhizosphere soil samples, six replicates from each of the three treatments (CK, R3 and Ya-1) were analyzed. Genomic DNA was extracted using the E.Z.N.A. DNA Kit (Omega Bio-tek, Norcross, GA, United States) following the manufacturer’s instructions. DNA concentration and purity were measured with TBS-380 fluorometer (Turner BioSystems, USA) and NanoDrop 2000 spectrophotometer (Thermo Fisher Scientific, USA), respectively. The quality of the DNA extracts quality was evaluated on a 1% agarose gel. DNA was fragmented to an average size of 350 bp using a Covaris M220 system (Gene Company Limited, China) for paired-end library construction. Paired-end sequencing was performed on an Illumina NovaSeq 6,000 platform (Illumina Inc., San Diego, CA, USA) at Majorbio Bio-Pharm Technology Co., Ltd., (Shanghai, China). Raw sequencing data from 18 samples have been deposited in the NCBI Sequence Read Archive (accession number: PRJNA1126323).

### 2.7 Metagenomic assembly and annotations of microbial genomes

Raw reads from high-throughput sequencing were filtered using the fastp software^1^ to remove the adapters and low-quality reads (Chen et al., 2018). Clean reads from each sample were assembled with MEGAHIT software (version 1.1.2) (Li et al., 2015). Based on assembled contigs, open reading frames (ORF) were predicted using MetaGene software for genes >100 bp (Noguchi et al., 2006). The non-redundant ORF set was generated with CD-HIT software, applying the following parameters: identity >90% and overlap >90% (Fu et al., 2012). Reads from different samples were compared with the non-redundant gene catalog (default parameter, 95% identity) using SOAPaligner software to calculate the abundance information of genes in the corresponding samples (Li et al., 2008). The non-redundant gene set was compared with the NR database using BLASTP (BLAST Version 2.2.28+, e-value cutoff: 1^−5^), and species annotations were derived from the taxonomic information in the NR database. Species abundance was calculated as the sum of gene abundances associated with each species (Altschul, 1997). Abundance profiles were constructed at each taxonomic level (Domain, Kingdom, Phylum, Class, Order, Family, Genus, and Species). Bioinformatics analysis was performed on the cloud platform of Majorbio Bio-Pharm Technology Co., Ltd.

### 2.8 Statistical analyses

The experimental data were analyzed using SPSS 27.0 (SPSS Inc., Chicago, IL. United States). Differences were analyzed using ANOVA and Duncan’s test (*p* < 0.05 or *p* < 0.01). Data were visualized with Origin Pro 2021 software (Northampton, MA 01060, United States).

## 3 Result

### 3.1 Antagonistic activity of the two strains against plant pathogen *F. Oxysporum*

**Figure 1:**
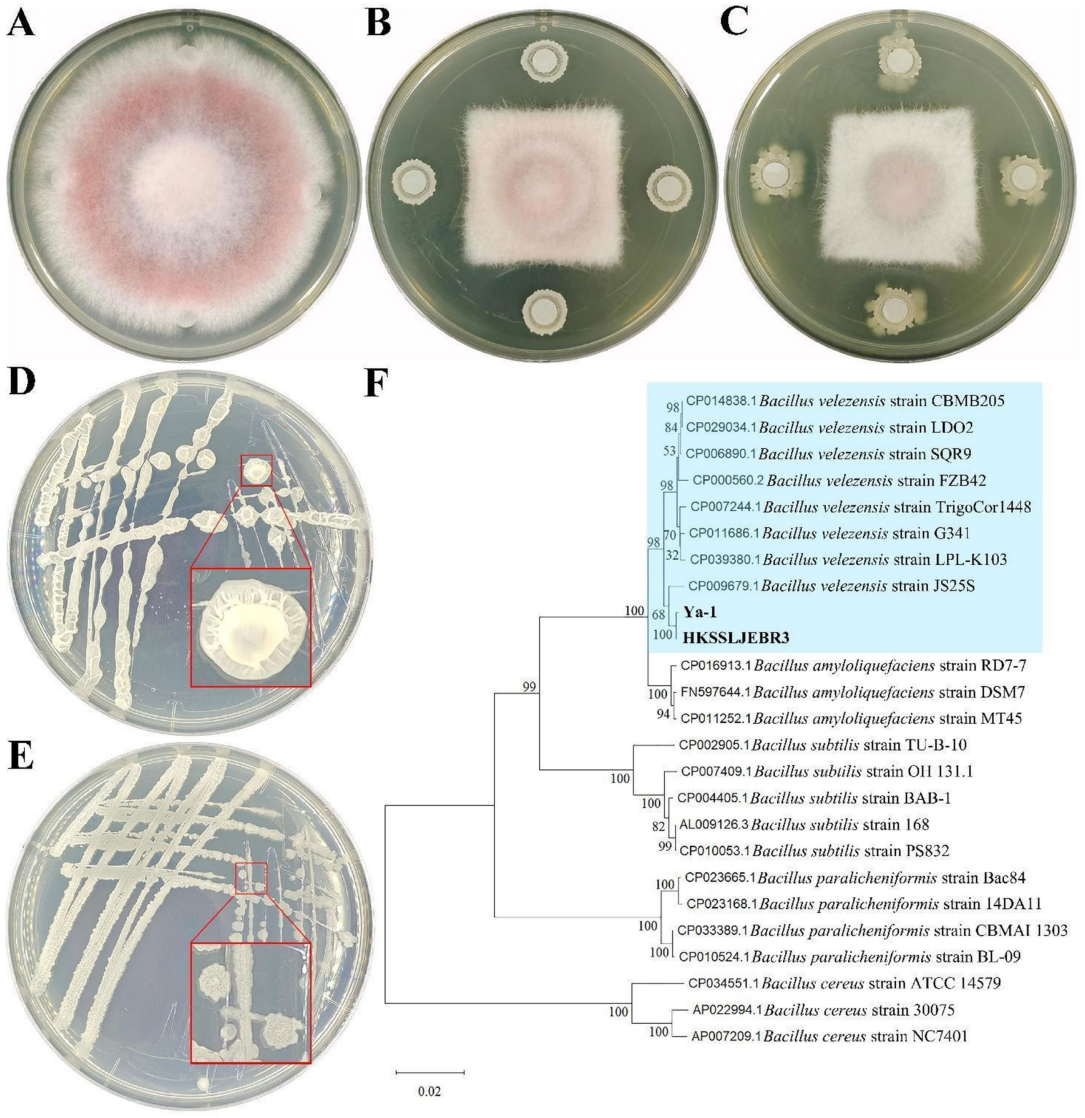
Inhibitory effects and identification of strains from different habitats. **(A)** *Fusarium oxysporum* CMTT4-1 and control treatment; **(B)** CMTT4-1 and R3 treatment; **(C)** CMTT4-1 and Ya-1 treatment. Treatments are shown after 7 days of cultivation. **(D)** R3 strain; **(E)** Strain Ya-1. The two strains are shown after 48 h of culture on LB plates. **(F)** Phylogenetic tree based on the sequences of the 16S rRNA and *gyrA* genes. The phylogenetic tree was constructed using the neighbor-joining method with MEGA 11. The numbers on the branches are the bootstrap support obtained from 1,000 replicates. Bold indicates the location of R3 and Ya-1 within the phylogenetic tree.

R3 and Ya-1 strains, isolated from pepper roots and tropical rainforest soil, respectively, were chosen as representative samples based on the result of preliminary screening. *In vitro*, assays demonstrated that both strains effectively hindered the growth of mycelia of *F. oxysporum*, with clearly defined inhibitory zones where neither the bacteria cultures nor the pathogen encroached upon one other. This led to a significant reduction in pathogen colony expansion compared to the control (Figures 1A–C). The inhibitory rates of R3 and Ya-1 against *F. oxysporum* were 48.54 ± 0.66% and 49.35 ± 1.44%, respectively. Therefore, both strains are potential candidates for the biological control of plant pathogenic fungi.

### 3.2 Morphological and molecular identification of the two strains

Morphological analyses revealed that R3 strain forms round or oval, light yellow, translucent colonies on LB plates 48 h after inoculation; the colonies were wet with uneven surfaces and irregular edges (Figure 1D). After 48 h of culture on LB plates, the Ya-1 strain formed light yellow opaque colonies in irregular patterns; the colonies were dry with uneven surfaces and irregular edges (Figure 1E).

For molecular identification, the 16S rRNA and *gyrA* genes were amplified via PCR and sequenced. The 16S rRNA gene sequences of both R3 and Ya-1 strains were 996 bp in length and shared a sequence identity of 99.80%. The sequences have been deposited in the GenBank database under accession numbers PP697635 (R3) and PP697636 (Ya-1). The *gyrA* gene sequences of both strains were 1,433 bp, with 100% sequence identity between them. These sequences are also available in GenBank under accession numbers PP700257 (R3) and PP700258 (Ya-1). Sequence alignment analysis showed high similarity between the two strains and *B. velezensis* JS25R, with sequence identities exceeding 99% for 16S rRNA and 98% for *gyrA*. A phylogenetic tree was constructed by concatenating both gene sequences and applying the NJ method, which clustered R3 and Ya-1 with eight other *B. velezensis* strains (JS25R, LPL-K103, G341, TrigoCor1448, FZB42, SQR9, LDO2, and CBMB205), confirming their classification within the *B. velezensis* species (Figure 1F). Additionally, a maximum-likelihood phylogenetic tree, constructed using MEGA software, corroborated the findings from the NJ method (Supplementary Figure S1). Based on the morphological and molecular analyses, R3 and Ya-1 were identified as *B. velezensis*.

### 3.3 Effects of inoculation with strains from different sources on pepper plant growth

**Figure 2:**
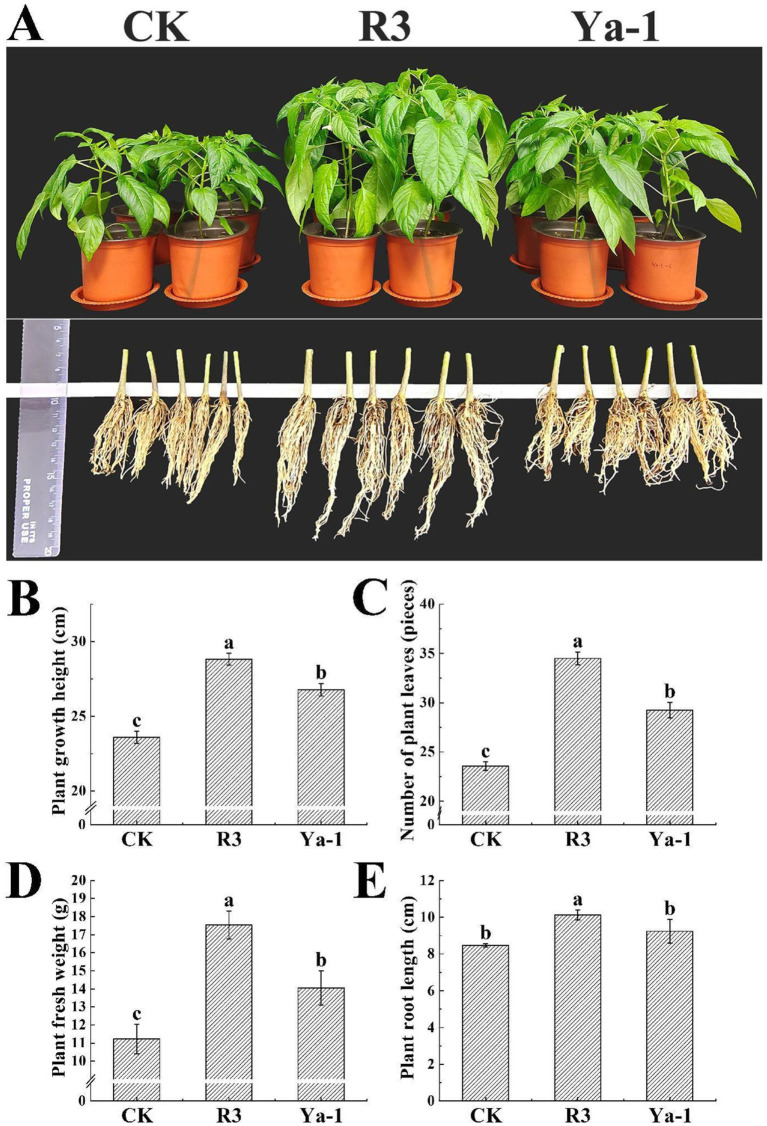
The strains from the different habitats promote the growth of pepper plants. **(A)** The effects of the R3 and Ya-1 microbial inoculants on peppers 20 days after the last treatment of 1 × 10^6^ CFU/mL (20 mL per plant). Pepper plants from each treatment group were randomly selected for photographic display. **(B)** Plant growth heights. **(C)** Number of plant leaves. **(D)** Plant fresh weights. **(E)** Plant root lengths. Different letters indicate significant differences between the different treatments at a confidence level of *p* < 0.05.

The effects of inoculation with the R3 and Ya-1 strains on the growth of pepper plants were evaluated. Twenty days following the final inoculation, both strains significantly promoted plant growth compared to the control treatment (Figure 2A). Specifically, R3 and Ya-1 strains increased the growth height of the pepper plants by 22.12 and 13.47%, respectively, relative to the control group. The number of leaves and the fresh weight of the plants also showed significant increases, with R3 and Ya-1 promoting leaf production by 46.44 and 24.13%, respectively, and increasing fresh weight by 56.29 and 27.17%, respectively (*p* < 0.05). Additionally, inoculation with the R3 resulted in a significant 19.47% increase in root length compared to the control group (*p* < 0.05; Figure 2). Overall, the R3 strain, isolated from the roots of chili peppers, demonstrated a significantly greater growth-promoting effect than the Ya-1 strain, which was isolated from tropical rainforest soil.

### 3.4 Effects of microbial inoculants on soil physicochemical properties

Inoculation with strains from different sources resulted in significant changes in the physicochemical properties of the soil. The R3 and Ya-1 strains increased the soil AK content by 5.13 and 57.34%, respectively, and enhanced the SOM content by 4.03 and 7.79%, respectively, compared to the control group (*p* < 0.05). However, R3 significantly reduced the soil AP content by 35.28% relative to the control group (*p* < 0.05). Neither R3 nor Ya-1 treatments significantly affected TN or TP contents when compared with the control group (*p* < 0.05; Table 1).

**Table 1:** The effects of microbial inoculants from different sources on soil properties.

	TN (g/kg)	TP (g/kg)	AP (mg/kg)	AK (mg/kg)	SOM (g/kg)
CK	6.04 ± 0.06a	1.39 ± 0.04ab	665.97 ± 28.16a	1429.93 ± 9.97c	665.40 ± 6.09c
R3	6.34 ± 0.16a	1.35 ± 0.04b	430.99 ± 45.57b	1503.28 ± 5.76b	692.19 ± 8.58b
Ya-1	6.17 ± 0.39a	1.43 ± 0.01a	620.96 ± 26.03a	2249.87 ± 0.13a	717.20 ± 12.06a

TN, total nitrogen; TP, total phosphorus; AP, available phosphorus; AK, available potassium; SOM, soil organic matter. Data followed by the different lowercase letters are significantly different at *p* < 0.05.

### 3.5 Metagenome analysis

A total of 815,583,476 raw sequence reads were obtained from 18 libraries with reads ranging from 40,592,958 to 55,870,300 per sample (Supplementary Table S1). After quality filtering, 797,390,216 clean reads were identified, and the percentage of clean reads relative to raw sequences per library exceeded 96.24% (Supplementary Table S2). Metagenome assembly yielded 12,386,282 contigs, containing a total of 8,307,576,614 bp (Supplementary Table S3). A total of 16,112,815 ORF were predicted, with an average ORF length of 454.57 bp, the longest being 31,572 bp and the shortest 102 bp (Supplementary Table S4). Based on aligning sequences against the NR database, the bacterial and fungal non-redundant gene sets were established. The bacterial gene catalog includes 4,652,315 genes with a total length of 2,349,773,235 bp and an average length of 505.08 bp. The fungal gene catalog consists of 37,957 genes with a total length of 25,200,180 bp and an average length of 663.91 bp.

### 3.6 Alpha diversity index and principal coordinate (PCoA) analysis of rhizosphere soil microbial communities

The effects of the two different *B. velezensis* strains (R3 and Ya-1) on rhizosphere soil microbial diversity were compared by evaluating their impact on the alpha diversity indices of the bacterial and fungal communities. These indices included the Ace and Chao indices for community richness, the Shannon and Simpson indices for community diversity, and the Coverage index to assess sequencing depth. The Coverage index was 1 for all samples, indicating sufficient sequencing depth for reliable diversity analysis. In the bacterial community, both the Ace and Chao indices were significantly higher in the R3 treatment compared to the other two treatments, indicating that R3 treatment resulted in a richer bacterial community. However, R3 treatment also resulted in a significantly lower Shannon index and a significantly higher Simpson index compared to the other two treatments, indicating reduced bacterial diversity. In the fungal community, the Ace and Chao indices were significantly higher in the Ya-1 treatment compared to the control, while the R3 treatment showed lower indices, indicating the highest fungal richness in the Ya-1 treatment and the lowest in the R3 treatment. Additionally, the Shannon index was significantly higher and the Simpson index was significantly lower in the Ya-1 treatment compared with the other two treatments, suggesting that Ya-1 treatment resulted in the highest fungal diversity, while R3 treatment induced the lowest fungal diversity (*p* < 0.05; Table 2).

**Table 2:** Alpha diversity analysis of microbial bacteria and fungi in pepper rhizosphere soil.

Microbial community	Treatment	Ace	Chao	Shannon	Simpson	Coverage
Bacteria	CK	3727.50 ± 25.60b	3727.50 ± 25.60b	5.14 ± 0.06a	0.0100b	1.00a
	R3	3789.50 ± 45.53a	3789.50 ± 45.53a	5.06 ± 0.05b	0.0200a	1.00a
	Ya-1	3724.00 ± 54.23b	3724.00 ± 54.23b	5.18 ± 0.05a	0.0100b	1.00a
Fungi	CK	485.17 ± 24.64b	485.17 ± 24.64b	1.38 ± 0.14b	0.5463 ± 0.0515b	1.00a
	R3	403.17 ± 19.27c	403.17 ± 19.27c	0.86 ± 0.27c	0.7584 ± 0.0853a	1.00a
	Ya-1	516.67 ± 20.65a	516.67 ± 20.65a	1.74 ± 0.08a	0.4120 ± 0.0256c	1.00a

Values are means ± SD (*n* = 6). Means sharing a common letter within the same column are not signifcantly different at *p* < 0.05.

**Figure 3:**
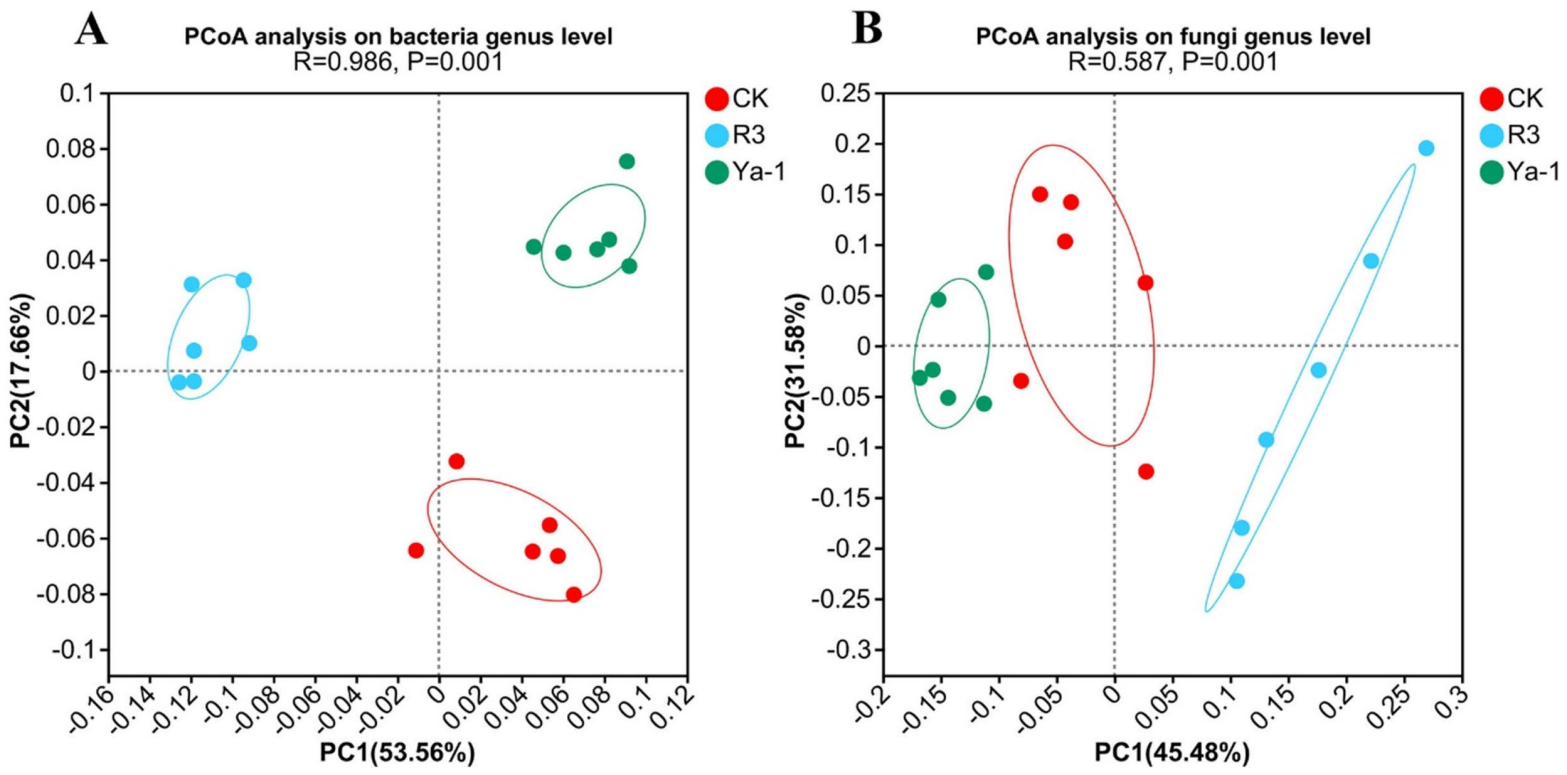
PcoA of bacterial **(A)** and fungi **(B)** communities in pepper rhizosphere soil on genus level.

PCoA based on Bray-Curtis distances was performed to analyze and visualize differences in microbial diversity among CK, R3 and Ya-1 treatments. The analysis revealed a clear separation of both bacterial and fungal communities in the R3 and Ya-1 treatments from the control, indicating significant differences in microbial community diversity among the three treatments (bacteria genus level, ANOSIM, *R* = 0.986, *p* = 0.001; fungi genus level, ANOSIM, *R* = 0.587, *p* = 0.001; Figure 3). Overall, these findings demonstrate that the source of *B. velezensis* strains has distinct effects on rhizosphere microbial communities.

### 3.7 Effects of R3 and Ya-1 strains on bacterial and fungal community composition

**Figure 4:**
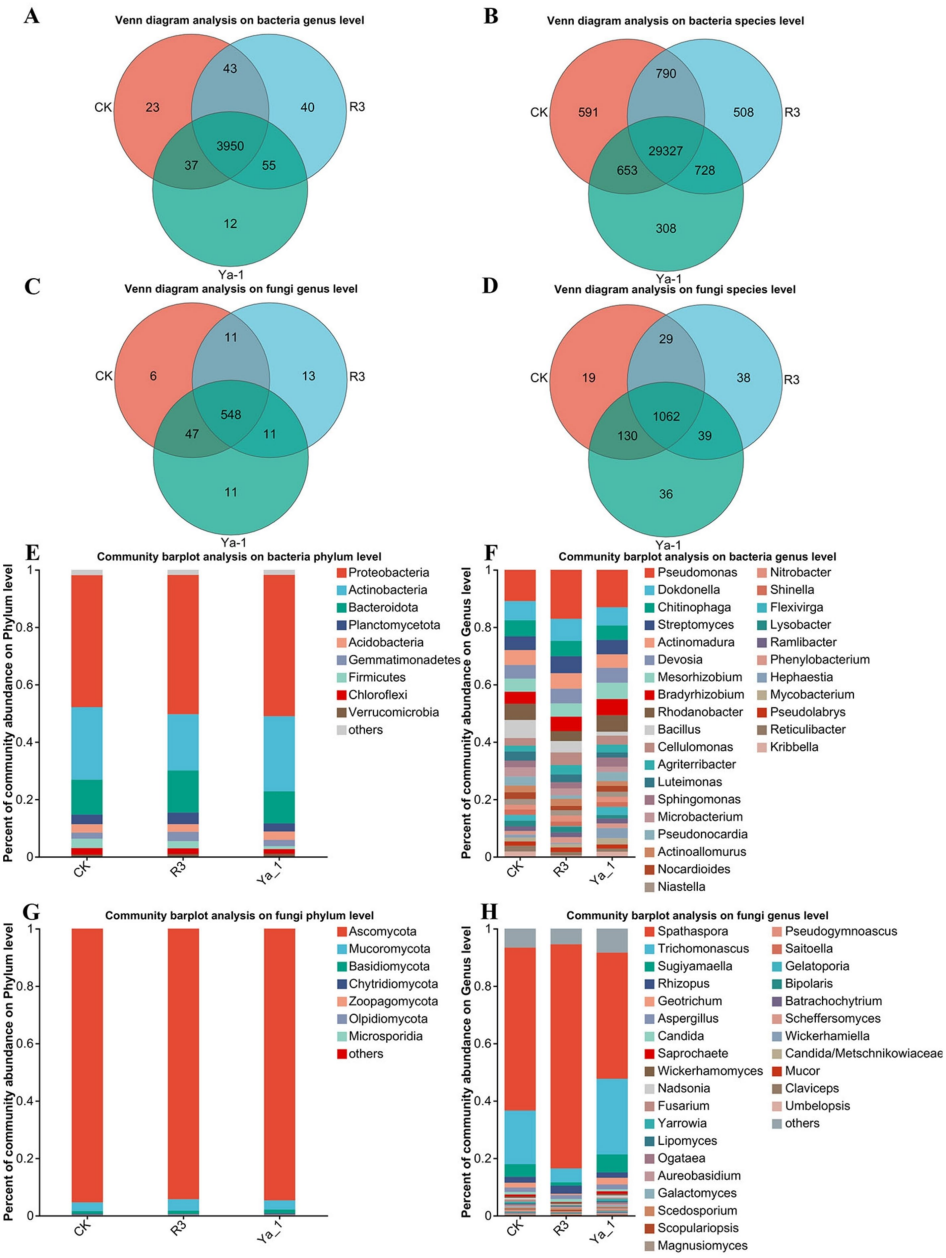
Effects of strains from different habitats on the number and composition of rhizosphere microorganisms. **(A)** Venn diagram analysis of the bacteria at the genus level; **(B)** Venn diagram analysis of the bacteria at the species level; **(C)** Venn diagram analysis of the fungi at the genus level; **(D)** Venn diagram analysis of the fungi at the species level; **(E)** Community bar plot analysis of the bacteria at the phylum level, top 9; **(F)** Community bar plot analysis of the bacteria at the genus level, top 30; **(G)** Community bar plot analysis of the fungi at the phylum level, top 7; **(H)** Community bar plot analysis of the bacteria a the genus level, top 30. The abscissa represents different treatments, and the ordinate represents the relative abundance of microorganisms.

Venn diagrams were utilized to depict the overlap and uniqueness of microorganisms between the CK group and *B. velezensis* inocula from different sources group treatments at the genus and species levels. At the bacterial genus level, the CK, R3 and Ya-1 groups contained 4,053, 4,088 and 4,058 genera, respectively. Of these, 3,950 genera were shared, with 23, 40 and 12 genera unique to the CK, R3 and Ya-1 groups, respectively (Figure 4A). At the bacterial species level, the CK, R3 and Ya-1 groups contained 31,361, 31,353 and 31,016 species, respectively, with 29,327 species shared across all groups and 591, 508 and 308 unique species in CK, R3 and Ya-1, respectively (Figure 4B). At the fungal genus level, the CK, R3 and Ya-1 groups comprised 612, 583 and 617 genera, respectively, with 548 genera shared and 6, 13 and 11 unique genera for each group (Figure 4C). At the fungal species level, the CK, R3 and Ya-1 groups contained 1,240, 1,168 and 1,267 species, respectively, with 1,062 shared and 19, 38 and 36 unique species in each group (Figure 4D).

A community bar plot was used to compare the microbial composition structure at the phylum and genus levels between the CK group and the *B. velezensis* inocula from different sources. At the bacteria phylum level, the dominant bacteria communities in the three treatments were similar, with Proteobacteria (CK: 45.99%, R3: 48.54% and Ya-1: 49.24%), Actinobacteria (CK: 25.26%, R3: 19.59% and Ya-1: 26. 19%), and Bacteroidota (CK: 12.17%, R3: 14.68% and Ya-1: 11.18%) (Figure 4E). The top 30 bacterial genera are shown in Figure 4F. In the CK group, the five most abundant genera were *Pseudomonas* (10.9%), *Dokdonella* (6.68%), *Bacillus* (6.31%), *Rhodanobacter* (5.61%), and *Chitinophaga* (5.58%). In the R3 group, the top five genera were *Pseudomonas* (17.08%), *Dokdonella* (7.69%), *Streptomyces* (5.93%), *Actinomadura* (5.38%), and *Chitinophaga* (5.31%). In the Ya-1 group, the five most abundant genera were *Pseudomonas* (13.06%), *Dokdonella* (6.34%), *Rhodanobacter* (5.73%), *Mesorhizobium* (5.67%), and *Bradyrhizobium* (5.63%). To summarize, R3 treatment significantly reduced the relative abundance of Actinobacteria at the bacterial phylum level when compared to the other two treatments. In contrast, both R3 and Ya-1 treatments increased the relative abundance of *Pseudomonas* relative to the CK treatment, with R3 showing a notably stronger effect.

The fungal community compositions were also similar across the three treatments, with the top three phyla being Ascomycota (CK: 95.35%, R3: 94.21% and Ya-1: 94.62%), Mucoromycota (CK:2.99%, R3: 4.00% and Ya-1: 3.21%) and Basidiomycota (CK: 1.02%, R3: 1.15% and Ya-1: 1.34%) (Figure 4G). The top 30 genera are presented in Figure 4H. In the CK group, the five most abundant genera were *Spathaspora* (56.78%), *Trichomonascus* (18.7%), *Sugiyamaella* (4.39%), *Rhizopus* (2.04%), and *Geotrichum* (1.69%). In the R3 group, the top five genera were *Spathaspora* (78.08%), *Trichomonascus* (4.84%), *Rhizopus* (2.83%), *Aspergillus* (1.39%), and *Sugiyamaella* (1.13%). In the Ya-1 group, the five most abundant genera were *Spathaspora* (43.98%), *Trichomonascus* (26.35%), *Sugiyamaella* (6.23%), *Geotrichum* (2.32%), and *Rhizopus* (1.88%). In summary, R3 treatment significantly increased the abundance of *Spathaspora* relative to the other two treatments. Both R3 and Ya-1 treatments also reduced the abundance of *Trichomonascus*, with R3 demonstrating a more significant effect.

**Figure 5:**
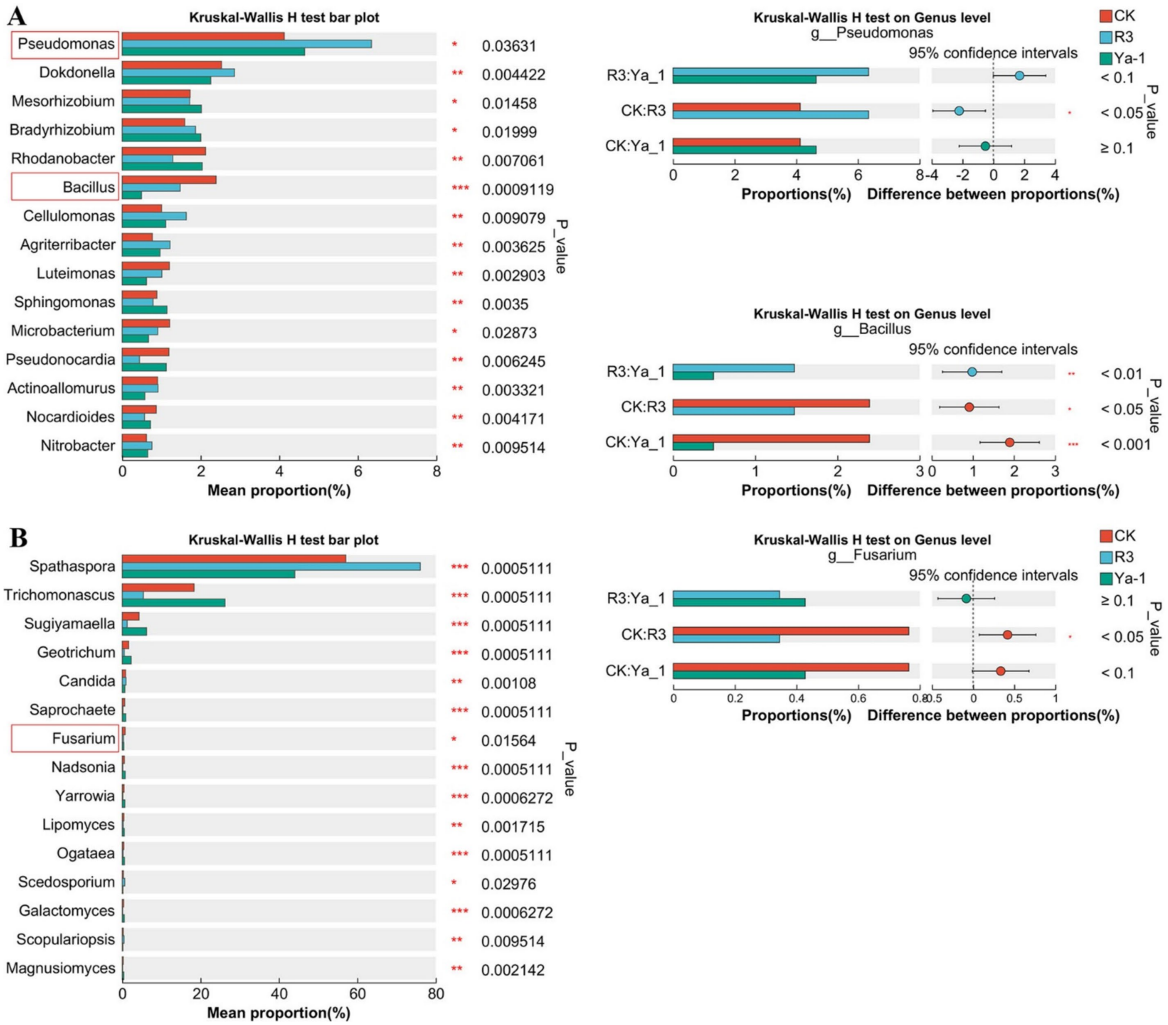
The groups were compared, and the top 15 microbial communities with significant differences are shown. **(A)** Kruskal–Wallis H analysis at the bacteria genus level. **(B)** Kruskal–Wallis H analysis at the fungi genus level. Paired comparisons of the three genera of *Pseudomonas*, *Bacillus*, and *Fusarium* between different treatments are shown on the right. *p < 0.05, ** *p* < 0.01, *** *p* < 0.001.

To evaluate the influence of rhizosphere microbial communities on plant growth, Kruskal–Wallis H tests were employed to compare microbial abundances at the genus level. The top 15 most significant differences are illustrated in Figure 5, after excluding the unclassified taxa. Both beneficial bacteria and pathogenic fungi within the rhizosphere were found to impact plant growth. At the bacterial genus level, *Pseudomonas* abundance was significantly higher in the R3 and Ya-1 groups, with a notable difference between the R3 and the CK group (*p* < 0.05). In contrast, *Bacillus* was significantly less abundant in the R3 and Ya-1 groups than in the CK group (Figure 5A). At the fungus genus level, *Fusarium* abundance was reduced in the R3 and Ya-1 groups, with R3 showing a significant difference compared to the CK group (*p* < 0.05).

### 3.8 Correlation between soil physicochemical property parameters and microbial community composition

**Figure 6:**
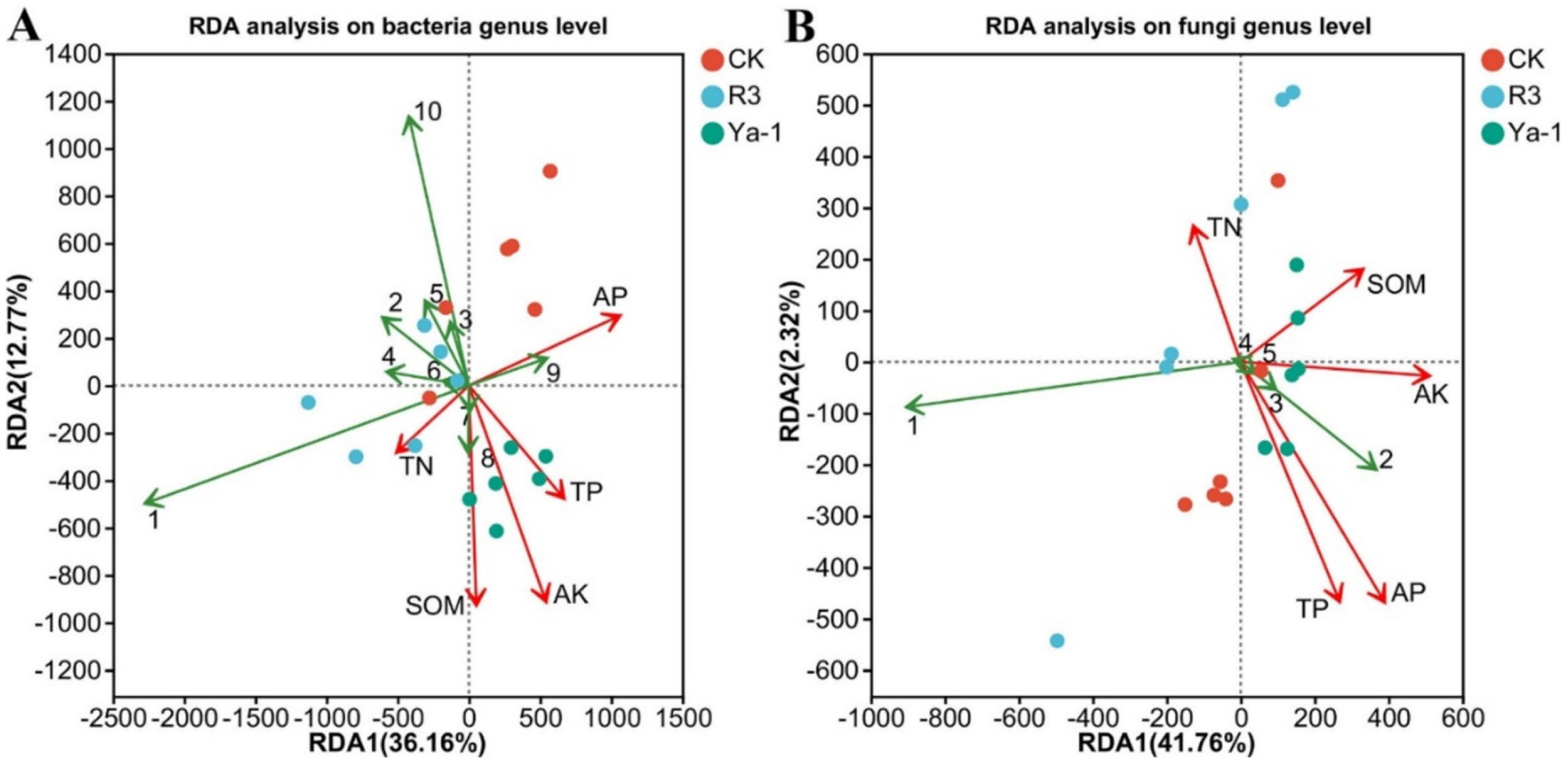
Redundancy analysis (RDA) of the soil physicochemical property parameters and microbial community composition. Soil physicochemical parameters are represented by red lines. **(A)** RDA at the bacteria genus level. Bacteria genera are represented by green lines (1: *Pseudomonas*, 2: *Dokdonella*, 3: *Chitinophaga*, 4: *Streptomyces*, 5: *Actinomadura*, 6: *Devosia*, 7: *Mesorhizobium*, 8: *Bradyrhizobium*, 9: *Rhodanobacter*, 10: *Bacillus*). **(B)** RDA at the fungi genus level. Fungi genera are represented by green lines (1: *Spathaspora*, 2: *Trichomonascus*, 3: *Sugiyamaella*, 4: *Rhizopus*, 5: *Geotrichum*). The lengths of the lines represent the degree of correlation between the environmental factors and genera. The longer the line, the greater the correlation, and vice versa. The angles between the environmental factors and the genera represent the positive and negative correlations between them (acute angle: positive correlation; obtuse angle: negative correlation; right angle: no correlation).

The distribution of samples was visualized and the relationship between microbial taxa and environmental variables were assessed through redundancy analyses. At the bacteria genus level, *Dokdonella*, *Chitinophaga*, *Streptomyces*, *Actinomadura*, *Devosia*, and *Bacillus* showed negative correlations with TP, AK, and SOM. In contrast, *Mesorhizobium* and *Bradyrhizobium* exhibited positive correlations with TN, TP, AK, and SOM (Figure 6A). At the fungal genus level, *Spathaspora* negatively correlated with TP, AP, AK, and SOM, while *Trichomonascus*, *Sugiyamaella* and *Geotrichum* were positively correlated with these same soil variables (Figure 6B). These results suggest that shifts in microbial community composition are instrumental in shaping soil physical and chemical properties.

## 4 Discussion

PGPM represents a promising alternative to chemical pesticides due to their effectiveness, environmental safety, and non-toxic nature (El-Saadony et al., 2022). PGPM can be sourced from diverse environments such as marine ecosystems, soil and plants (Al Hamad et al., 2021; De Souza et al., 2021; Yang et al., 2024). However, the impact of microorganisms on plant growth may vary significantly depending on their habitat and environmental conditions. To explore this variability, the R3 and Ya-1 strains were isolated from the roots of pepper plants and tropical rainforest soil, respectively. Both strains were identified as *B. velezensis* through morphological characterization and molecular analysis of 16S rRNA and *gyrA* genes. *In vitro*, dual-culture assays demonstrated that both strains exhibited strong antagonistic activity against plant pathogens, consistent with findings reported by Tan et al. (2015). This inhibitory effect is likely attributed to the production of bioactive secondary metabolites with antifungal properties. Overall, the results indicate that the R3 and Ya-1 strains have the potential to control plant diseases.

Microorganisms contribute to plant growth via both direct and indirect mechanisms (El-Saadony et al., 2022). Direct mechanisms include the production of plant hormones, nutrient dissolution, synthesis of antibacterial substances, secretion of cell wall-degrading enzymes, biofilm formation, and extracellular polysaccharide production, whereas the indirect mechanisms involve activating antioxidant enzymes, inducing systemic resistance, accumulating osmotic protectants, enhancing plant nutritional status, regulating internal signal transduction, reducing heavy metal stress, and improving water uptake and utilization (Elnahal et al., 2022; Fadiji et al., 2022; Kumawat et al., 2022; Singh et al., 2022). Previous research has shown that *B. velezensis* CE100 suppresses pathogen growth while producing indoleacetic acid (IAA), which promotes growth in strawberry plants (Hong et al., 2022). Similarly, *B. velezensis* LY7 has been reported to promote growth and induce resistance to *Colletotrichum scovillei* (Zou et al., 2024). In this study, the R3 and Ya-1 strains, isolated from different environments, both promoted growth in pepper plants, as evidenced by increased plant height, leaf number, fresh weight and root length (Figure 4). These results are consistent with previous studies by Tian et al. (2023) and Shi et al. (2022). The growth-promoting effect of the R3 strain was more pronounced, possibly due to its endophytic origin in pepper plant roots, which may confer better adaptation to the host plant compared to the Ya-1 strain, which was isolated from tropical rainforest soil. This supports the idea that host-endophytic bacteria interactions are typically mutualistic, where the plant offers nutrients and protection to the bacteria, while the bacteria produce metabolites such as IAA and nitrogen-fixing enzymes, which enhance plant growth. For instance, *B. paralicheniformis* from rice roots can fix nitrogen (Annapurna et al., 2018) and endophytic bacteria from *Corchorus olitorius* produce IAA (Haidar et al., 2018), thereby promoting plant growth.

This study demonstrates that microbial inoculants R3 and Ya-1 significantly improve the physical and chemical properties of soil, particularly in terms of increasing AK and SOM content when compared to the control group. Interestingly, soil treated with R3 exhibited a lower AP content than the other treatments, suggesting that R3 may facilitate the solubilization of phosphorus. This hypothesis is further supported by the observation that TP content was lower in R3-treated soils compared to Ya-1-treated soils. However, further studies are required to conclusively confirm the phosphorus-dissolving mechanisms associated with R3. The ability of *B. velezensis* to solubilize phosphorus is well established, and strains such as QA2 and Ag75 have been shown to enhance the growth of quinoa, maize, and soybean by increasing the bioavailability of phosphorus (Mahdi et al., 2022; Mosela et al., 2022). These findings support the idea that R3 may promote plant growth through similar mechanisms, potentially enhancing phosphorus uptake by plants. Furthermore, the results presented in Section 3.8 indicate that specific physicochemical properties of soil are closely linked to the microbial communities present, a finding consistent with the work of Shi et al. (2022). This interaction between soil properties and microbial communities is likely a key factor in promoting the growth of pepper plants in our study. Soil microorganisms are highly responsive to nutrient availability, particularly N, P and K, which are known to influence microbial activity and community composition (Allison and Martiny, 2008). Notably, the relationship between arbuscular mycorrhizal fungi and soil nutrient content has been well documented, with these fungi showing a negative correlation with phosphorus levels and a positive correlation with nitrogen content (Koorem et al., 2014). Additionally, many microorganisms exhibit a positive relationship with SOM content (Dasgupta and Brahmaprakash, 2021), highlighting the importance of organic matter in fostering a diverse and active microbial community.

Rhizosphere microorganisms has been termed the “secondary genome” of plants due to its critical role in plant development and stress resistance (Kumar and Dubey, 2020). Metagenome sequencing provides us with a means to understand rhizosphere microbiota as it can capture all the genomic information in a specific environment. Previous studies have shown that inoculation with *B. amyloliquefaciens* B1408 alters microbial alpha diversity, and PCoA analysis can clearly reveal the differences in microbial community structure among different treatments (Han et al., 2019). In this study, inoculation with *B. velezensis* strains R3 and Ya-1, isolated from distinct habitats, also led to significant shifts in the alpha diversity of the rhizosphere microbial community. PCoA analysis further supported these findings, clearly distinguishing microbial communities across different treatment groups. Venn diagram analysis at both the genus and species levels revealed that prokaryotes predominated in the rhizosphere, with eukaryotes representing a secondary component (Figure 6). The dominant bacterial phyla included Proteobacteria, Actinobacteria, Bacteroidota, Planctomycetota, and Acidobacteria. Eukaryotic members of the rhizosphere were primarily represented by Ascomycota, Mucoromycota, and Basidiomycota at the phylum level. These findings are in agreement with previous research on rhizosphere microbiota composition (Mohanram and Kumar, 2019). Collectively, our results indicate that inoculation with *B. velezensis* strains R3 and Ya-1 significantly alters the microbial community structure of the rhizosphere, highlighting the potential of these inoculants to modulate soil microbial dynamics for enhanced plant growth.

The top 15 genera were significantly different between the multiple groups (Figure 6). In this study, we explored the dual role of microbes in influencing plant growth, focusing on the beneficial effects of PGPM and the detrimental effects of plant pathogens. *Pseudomonas*, a well-known PGPM, has been shown to suppress plant pathogens and promote plant growth (Bonaterra et al., 2022; Mehmood et al., 2023), while *Fusarium*, a stubborn soil-borne pathogen, produces harmful secondary metabolites, including mycotoxins, which contribute to plant wilt and yield reduction (Ma et al., 2013; Perincherry et al., 2019). In our results, *Pseudomonas* abundance was highest in the R3 treatment group with previous studies. For instance, Sun et al. demonstrated that the inoculation of *B. velezensis* SQR9 could enhance *Pseudomonas* populations in the cucumber rhizosphere (Sun et al., 2022), supporting our observation. Additionally, *Fusarium* abundance in the R3 and Ya-1 groups was lower than that in the CK group, suggesting an inhibitory effect on *Fusarium* colonization. This conclusion was supported by the results of the double-culture confrontation assays, which demonstrated antagonistic interactions between the inoculated strains and *Fusarium*. These results demonstrate that inoculation with R3 can recruit beneficial microorganisms while restricting microorganisms that are unfavorable to pepper plants. Interestingly, the abundance of *Bacillus* in the R3 and Ya-1 groups was significantly lower than that in the CK group. The inoculant may have dynamically changed over time in the plant rhizosphere, but the differences were only measured on the 20th day after inoculation. Thus, future investigations should focus on changes in microbial communities at different time intervals, after microbial inoculation.

## 5 Conclusion

This study investigates the potential of two *B. velezensis* strains, R3 and Ya-1, isolated from different environments, as PGPMs for pepper plants. Both strains exhibited strong antagonistic activity against plant pathogens and promoted pepper plant growth, particularly R3, which showed a more pronounced effect due to its endophytic origin. The inoculants improved soil properties, including increased AK and SOM content, and influenced microbial community dynamics in the rhizosphere, with notable shifts in microbial diversity. In particular, R3 enhanced beneficial microorganisms like *Pseudomonas* while inhibiting harmful pathogens such as *Fusarium*. The results highlight the potential of these PGPMs for sustainable plant growth promotion and disease control, although further studies are needed to confirm mechanisms such as phosphorus solubilization.

## Data Availability

The datasets presented in this study can be found in online repositories. The names of the 506 repository/repositories can be found at: https://www.ncbi.nlm.nih.gov/bioproject/PRJNA1126323/. The accession numbers of sequenced raw reads are provided in Supplementary Table S1.

## Footnotes

^1^ https://github.com/OpenGene/fastp
