# Supplementary figures and images for: Effects of two Bacillus velezensis strains isolated from different sources on the growth of Capsicum annum
Source: Front Microbiol. 2024 Dec 9;15:1504660. doi: 10.3389/fmicb.2024.1504660 (PMC11663850; doi:10.3389/fmicb.2024.1504660)

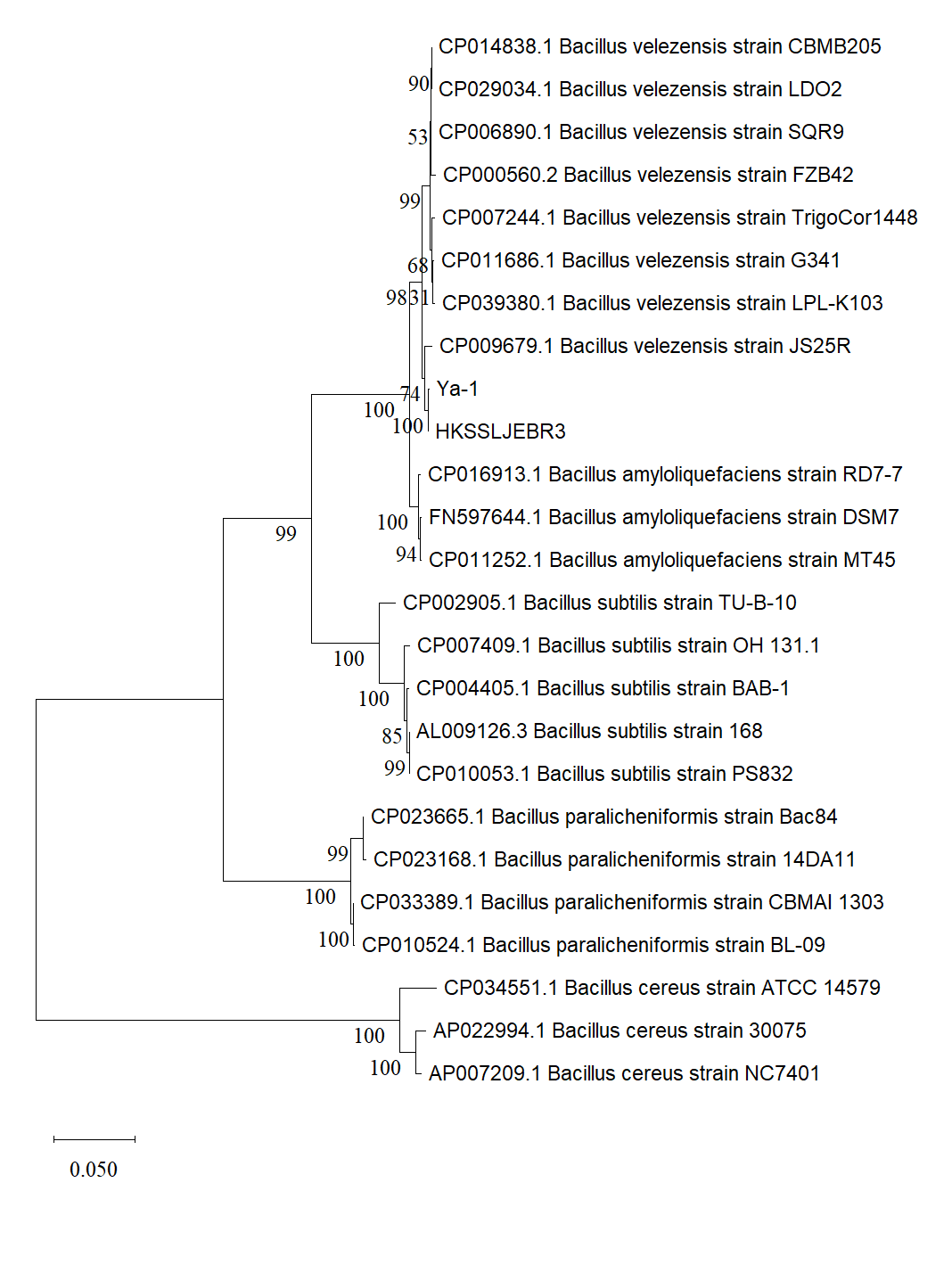

Supplement: Supplementary file 1 [file Image_1.tif]
